# Evaluation of the VITEK MS knowledge base version 3.0 for the identification of clinically relevant *Mycobacterium* species

**DOI:** 10.1038/s41426-018-0120-3

**Published:** 2018-07-04

**Authors:** LiuLin Luo, Wen Cao, WeiWei Chen, RanRan Zhang, LinJie Jing, HuiPing Chen, FangYou Yu, Jun Yue

**Affiliations:** 1 Department of Clinical Laboratory Medicine, Shanghai Pulmonary Hospital, Tongji University School of Medicine, Shanghai, 200433 China; 2 Tongji University School of Medicine, Shanghai, 200092 China

## Abstract

Different *Mycobacterium* spp. infections may indicate varied treatment regimens in the clinic. Thus, the species-level identification of *Mycobacterium* spp. is one of the most important tasks for a clinical microbiology laboratory. Although matrix-assisted laser desorption ionization–time of flight mass spectrometry (MALDI-TOF MS) has emerged as a rapid and accurate method for the identification of mycobacteria, this method lacks a comprehensive evaluation of the identification accuracy for clinically collected mycobacteria using VITEK MS Knowledge Base Version 3.0 (Ver 3.0). The objectives of the present study were to evaluate the identification performance of *Mycobacterium* spp. using Ver 3.0 and a sample processing kit for strain inactivation and protein extraction. Among the 507 *Mycobacterium* isolates, 46 isolates were *M. tuberculosis*, and 461 isolates were nontuberculous mycobacteria (NTM) (including 27 species: 17 species were slowly growing mycobacteria (SGM), and 10 species were rapidly growing mycobacteria (RGM)). The VITEK MS V3.0 library was used to correctly identify 476/507 (93.9%) isolates (425 isolates were correctly identified initially, and 51 more isolates were correctly identified on repeat), 23/507 (4.5%) isolates were unidentified, and 8/507 (1.6%) isolates were misidentified. In summary, we showed that *Mycobacterium* spp. can be adequately identified by Ver 3.0 in combination with the use of a standard sample processing kit.

## Introduction

*Mycobacterium* spp. comprise a large group of microorganisms, and many species within the genus are prominent pathogens, particularly the *M. tuberculosis* complex (MTBC)^1^. Tuberculosis (TB) caused by MTBC is the ninth leading cause of death worldwide and the leading cause from a single infectious agent, ranking above HIV/AIDS, with 10.4 million illnesses and 1.8 million deaths reviewed in the 2016 WHO report^2^. Apart from MTBC, many NTM (nontuberculous mycobacteria) species are also sources of important diseases in humans^3,4^. These species are considered important opportunistic pathogens that can cause pulmonary, skin, soft tissue, and disseminated infections^5–7^.

The identification of *Mycobacterium* at the species level is not only important to discriminate true pathogens from environmental contaminants but also to reveal differences in clinical relevance and treatment regimens between NTM species^8,9^. Traditionally, *Mycobacterium* spp. have been identified using phenotypic methods^10^. However, these methods are laborious and cumbersome and may require long incubation periods. These procedures cannot meet the demands of clinical requirements that require prompt and accurate identification results. Thus, rapid, accurate identification of *Mycobacterium* is an important task of the clinical mycobacteriology laboratory^9^.

The use of molecular techniques has dramatically accelerated the *Mycobacterium* diagnostic process. DNA probes and PCR-based detection have been available for many years and show good performance for certain species but show cross-reactions with *M. intracellulare* and *M. fortuitum* species, along with the *M. avium* complex^11–15^. Currently, the most reliable methods for the identification of all *Mycobacterium* spp. involve the molecular analysis of selected genes, such as 16 S *rRNA*, *rpoB*, and *hsp65*^16^. However, these techniques are expensive, technically complex, time-consuming, require specific equipment and expertise and have limited availability.

Matrix-assisted laser desorption ionization–time of flight mass spectrometry (MALDI-TOF MS) has revolutionized the identification of microorganisms and has recently been applied for the identification of *Mycobacterium* spp^17,18^. Many reports adopt this technology for the identification of *Mycobacterium* spp., but the results have been greatly varied^18–25^. Due to the presence of mycolic acids and the pathogenicity of mycobacteria, reliable inactivation and reproducible sample extraction protocols present great challenges, which might partly explain the great variances in the findings among researchers^17,26,27^. Another explanation may be the coverage of *Mycobacterium* spp. included in different commercial database platforms^19,22,28,29^.

The objective of the present study was to evaluate the identification performance of clinical *Mycobacterium* spp. using the VITEK MS Knowledge Base Version 3.0 in combination with the VITEK^®^ MS *Mycobacterium/Nocardia* Kit (bioMérieux SA, Marcy L’Étoile, France) to generate highly reliable results in sample processing.

## Results

### Inactivation results

To ensure the complete inactivation of *Mycobacterium*, we tested 10 isolates, including four MTBC and six NTM isolates. As expected, these pathogens were nonviable after a 10-min incubation in 70% ethanol following selection with a 1-µL inoculation loop after inactivation. Neither the MGIT nor the LJ medium made any difference in viability for any of the isolates tested after 6 weeks of incubation.

### VITEK MS identification

A total of 507 *Mycobacterium* isolates, comprising 28 species, were used for analysis. The 28 species included 17 SGM, 10 RGM, and 1 MTBC species. Among the 507 *Mycobacterium* isolates, 46 isolates were MTBC, 379 isolates were SGM, and 128 isolates were RGM. SGM was divided into 68 photochromogens (including *M. kansasii*), 39 scotochromogens (including *M. scrofulaceum*, *M. szulgai, M. colombiense*, *M. parascrofulaceum, M. lentiflavum* and *M. gordonae*), and 226 nonchromogens (including *M. avium*, *M. chimaera, M. timonense, M. intracellulare, M. arupense, M. florentinum, M. marseillense, M. xenopi, M. celatum*, and *M. gastri*).

MTBC is the most important pathogen among *Mycobacterium* spp., and the correct identification rate was 45/46 (97.8%). *Mycobacterium avium* and *M. intracellulare*, two important SGM pathogens, were correctly identified at rates of 54/56 (96.4%) and 150/153 (98.0%), respectively. For *M. kansasii*, the identification rate was 66/68 (97.1%). The following SGM species were less frequently encountered: *M. gordonae* 18/18 (100%), *M. lentiflavum* 8/8 (100%), *M. scrofulaceum* 5/5 (100%), *M. szulgai* 4/4 (100%), and *M. xenopi* 2/2 (100%). For RGM, *M. abscessus* and *M. fortuitum* are the two most frequently encountered species, with correct identifications at 88/90 (97.8%) and 16/18 (88.9%), respectively. All of the *M. abscessus* subspecies *abscessus* and *massiliense* were identified as *M. abscessus*. Regarding the *M. fortuitum* group, the species *M. fortuitum*, *M. senegalense*, *M. peregrinum* and *M. septicum* were all identified as *M. fortuitum* group. The RGM species *M. mucogenicum* 5/5 (100%), *M. mageritense* 1/1 (100%), *M. vaccae* 1/1 (100%), and *M. chelonae* 8/8 (100%) were also well identified.

During the VITEK MS Ver 3.0 evaluation, 425/507 (83.8%) isolates were initially identified, and 51/507 (10.1%) additional isolates were identified on repeat, with 23/507 (4.5%) isolates remaining unidentified and 8/507 (1.6%) isolates misidentified. All results are shown in Table 1.

**Table 1:** Performance of the Vitek MS version 3.0 library used for the identification of *Mycobacterium* spp.

	NO. (%) of isolates identified					
	Total	version 3.0	1^a^	2^b^	No ID^c^	Mis ID^c^
*M. tuberculosis*	46	45(97.8%)	40	5	1	0
*M. avium*	56	54(96.4%)	50	4	2	0
*M. intracellulare*	153	150(98.0%)	143	7	3	0
*M. marseillense*	6	0(0.0%)	0	0	6	0
*M. timonense*	1	0(0.0%)	0	0	0	1
*M. chimaera*	1	0(0.0%)	0	0	0	1
*M. colombiense*	2	0(0.0%)	0	0	2	0
*M. kansasii*	68	66(97.1%)	58	8	2	0
*M. gastri*	4	0(0.0%)	0	0	0	4
*M. abscessus*						
*M. abscessus sub abscessus*	71	70(98.6%)	55^f^	15	1	0
*M. abscessus sub massiliense*	19	18(94.7%)	11^f^	7	1	0
*M. fortuitum* group						
*M. fortuitum*	18	16(88.9%)	15^e^	1	2	0
*M. peregrinum*	1	1(100.0%)	1^e^	0	0	0
*M. septicum*	1	1(100.0%)	1^e^	0	0	0
*M. senegalense*	1	1(100.0%)	1^e^	0	0	0
*M. celatum*	1	1(100.0%)	1	0	0	0
*M. arupense*	1	1(100.0%)	1	0	0	0
*M. chelonae*	8	8(100.0%)	7	1	0	0
*M. florentinum*	1	0(0.0%)	0	0	1	0
*M. gordonae*	18	18(100.0%)	16	2	0	0
*M. lentiflavium*	8	8(100.0%)	8	0	0	0
*M. mageritense*	1	1(100.0%)	1	0	0	0
*M. mucogenicum*	5	5(100.0%)	4	1	0	0
*M. scrofulaceum*	5	5 (100.0%)	5^d^	0	0	0
*M. szulgai*	4	4(100.0%)	4	0	0	0
*M. vaccae*	1	1(100.0%)	1	0	0	0
*M. xenopi*	2	2(100.0%)	2	0	0	0
*M. monacense*	2	0(0.0%)	0	0	2	0
*M. parascrofulaceum*	2	0(0.0%)	0	0	0	2
Total	507	476	425	51	23	8
		93.9%	83.8%	10.1%	4.5%	1.6%

^a^Number of strains identified for the first time

^b^Number of strains identified for the second time

^c^ID, identification

^d^One of *M. scrofulaceum* was identified with a 50% confidence value for both *M. scrofulaceum* and *M. intracellulare*

^e^These species were all identified as *M. fortuitum* group

^f^These subspecies were all identified as *M. abscessus* in VITEK MS V3.0 database

### Unidentified and misidentified Isolates by VITEK MS

A total of 23 isolates were unidentified by the VITEK MS Ver 3.0 system. Among these species, the 11 isolates not in the database included 1 *M. florentinum*, 6 *M. marseillense*, 2 *M. colombiense*, and 2 *M. monacense*. The other 12 unidentified isolates included 1 MTBC, 3 *M. intracellulare*, and 2 each of *M. avium*, *M. kansasii*, *M. abscessus*, and *M. fortuitum* isolates. There were no misidentifications at the genus level when using the VITEK MS Ver 3.0 database. At the species level, 8 isolates were misidentified. Misidentifications were observed for 4 isolates of *M. gastri*, which were identified as *M. kansasii*, 2 *M. parascrofulaceum* isolates were identified as *M. scrofulaceum* and 1 isolate each of *M. timonense* and *M. chimaera* was identified as *M. intracellulare*. For other species, one *M. scrofulaceum* isolate was identified with a 50% confidence value for both *M. scrofulaceum* and *M. intracellulare*. The results are shown in Table 2.

**Table 2:** Analysis of the discrepancy strains

Sequencing result (N^a^)	Ver 3.0 Identification^b^	Ver 3.0 database^c^	Target sequenced^d^
*M. tuberculosis* (1)	No ID	YES	16 S rRNA
*M. avium* (2)	No ID	YES	16 S rRNA
*M. intracellulare* (3)	No ID	YES	16 S rRNA
*M. colombiense* (2)	No ID	NO	16 S rRNA
*M. marseillense* (6)	No ID	NO	16 S rRNA
*M. chimaera* (1)	*M. intracellulare*	NO	16 S rRNA
*M. timonense* (1)	*M. intracellulare*	NO	16 S rRNA
*M. kansasii* (2)	No ID	YES	16 S rRNA and *hsp65*
*M. gastri* (4)	*M. kansasii*	YES	16 S rRNA and *hsp65*
*M. abscessus subsp abscessus* (1)	No ID	YES	16 S rRNA and *erm*
*M. abscessus subsp massiliense* (1)	No ID	YES	16 S rRNA and *erm*
*M. fortuitum* (2)	No ID	YES	16 S rRNA and *hsp65*
*M. peregrinum* (1)	*M. fortuitum* group	YES	16 S rRNA and *hsp65*
*M. septicum* (1)	*M. fortuitum* group	NO	16 S rRNA and *hsp65*
*M. senegalense* (1)	*M. fortuitum* group	YES	16 S rRNA and *hsp65*
*M. florentinum* (1)	No ID	NO	16 S rRNA
*M. monacense* (2)	No ID	NO	16 S rRNA
*M. scrofulaceum* (1)	*M. scrofulaceum/M. intracellulare*	YES	16 S rRNA
*M. parascrofulaceum* (2)	*M. scrofulaceum*	NO	16 S rRNA

^a^N: the number of *Mycobacterium* strains obtained by the VITEK MS V3.0 database different from sequence results

^b^No ID: No identification

^c^species included in VITEK MS Ver 3.0 knowledge database or not

^d^*hsp65*: Sequence of the 441-bp Telenti fragment

## Discussion

To our knowledge, this study represents the most comprehensive evaluation of the CE/IVD and FDA-approved VITEK MS V3.0 for the identification of clinically isolated mycobacteria. The correct identification rate of *Mycobacterium* spp. after retesting was 93.9%. In addition, no isolates were misidentified at the genus level through VITEK MS V3.0.

MALDI-TOF MS is widely used for routine bacterial identification in clinical laboratories. Unlike bacteria and yeasts, mycobacteria have thicker cell walls and thus require an additional protein extraction procedure to ensure effective identification. Although many methods have been described, and perfect inactivation effects were obtained^18,21^, there is still a lack of uniform procedures for strain inactivation and protein extraction. Here, we used a kit from bioMérieux for these purposes.

MTBC remains the most important group within the genus *Mycobacterium* from a global and clinical perspective^2^. In the present study, 1/46 (2.2%) isolates were unidentified using the VITEK MS V3.0 analysis. This excellent identification performance with MTBC indicated that MALDI-TOF MS could be used as a powerful clinical tool. For SGM, the most frequently involved pathogens in human diseases were *Mycobacterium intracellulare* and *Mycobacterium avium*^30^. In the present study, only 2/56 (3.6%) *M. avium* and 3/153 (2%) *M. intracellulare* were unidentified; these rates are lower than the previously reported rates between 7.7 and 27.8%^17,31^. *Mycobacterium colombiense*, *M. chimaera*, *M. marseillense* and *M. timonense* are members of species closely related to *M. avium* and *M. intracellulare* but absent from the VITEK MS V3.0 database^32,33^. During the VITEK MS V3.0 analysis, all *M. colombiense* and *M. marseillense* strains were not identified, but *M. chimaera* and *M. timonense* were misidentified as *M. intracellulare*. This misidentification may be due to the close phylogenetic relationships between *M. intracellulare* and these two species^34,35^. *Mycobacterium kansasii* is second in frequency to *M. avium* and *M. intracellulare* as a cause of NTM lung disease, and its response to chemotherapy is much better^5^. Although the present results showed that the identification performance of *M. kansasii* is excellent, the major problem is that *M. gastri* was readily misidentified as *M. kansasi*. One reason for this misidentification is that incubation conditions may lead to excessive polymer production, which could greatly interfere with the spectra acquisition during MALDI-TOF MS analysis, while other explanations may be attributed to the genetic similarity between *M. kansasii* and *M. gastri*, as well as the potential that the entire range of strain diversity is not covered in the databases. However, these two species were also easy to interchange and may still need more spectra incorporated in the database^22,29,36–38^. Other SGM species involved in pulmonary diseases were all correctly identified (including *M. xenopi*, *M. lentiflavum* and *M. szulgai*)^4,39^. As expected, one isolate of *M. florentinum* not included in the database was unidentified. Except for the species not included in the database, human disease-related SGM species were well identified.

*Mycobacterium gordonae*, an SGM species that rarely causes human diseases, is a frequent clinical isolate, and all of these isolates were correctly identified^40^. Other SGM species mainly isolated from the respiratory tract but rarely associated with human diseases, such as *M. parascrofulaceum*, *M. celatum*, and *M. arupense*, were also included in the present study^41–43^. These findings were also subjected to the close relationships and limited spectra included in the knowledge database; one isolate of *M. scrofulaceum* was not well identified, while two isolates of *M. parascrofulaceum* were misidentified as *M. scrofulaceum*^44^. For the limited numbers of some species, such as *M. celatum* and *M. arupense*, 100% correct identifications were also observed. In addition to human disease-related SGM species, these rare SGM species also showed good identification performance in VITEK MS V3.0. Evaluation of more strains of these rare species could provide useful information.

The RGM are opportunistic pathogens that produce disease in a variety of clinical settings^39^. The three major clinically important species of RGM, *M. abscessus*, *M. fortuitum* group and *M. chelonae*, are responsible for approximately 80% of RGM-related mycobacterial diseases^7^. The *M. abscessus* complex, consisting of three subspecies (*M. massiliense*, *M. bolletii*, and *M. abscessus*), is the most common of the RGM species that cause NTM pulmonary diseases. Among these species, *M. abscessus* subsp *abscessus* is the dominating subspecies and is responsible for treatment failures, mainly due to the inducible resistance of an *erm* (position 28: C to T) mutation, which results in clarithromycin resistance, which would be impossible in *M. abscessus* subsp *massiliense* due to its incomplete *erm* gene^45,46^; thus, different subspecies of *M. abscessus* infections may result in varied therapies^47^. However, although the correct identification rate was perfect for *M. abscessus* (the unidentified two isolates are smooth-growing, one each of *M. abscessus* subspecies *abscessus* and *massiliense*), these subspecies could not be discriminated in the VITEK MS V3.0 knowledge database. In prospect, these subspecies should be differentiated by the VITEK MS IVD (In Vitro Diagnosis) system, owing to the different treatment requirements. *M. fortuitum* group includes up to 13 species, but the VITEK MS V3.0 knowledge database includes the seven most prominent and closely related species. Species such as *M. peregrinum*, *M. senegalense* and *M. septicum*, were all identified as the *M. fortuitum* group. Except for *M. septicum* species, *M. peregrinum* and *M. senegalense* were included in the database, which may be partially explained by the limited strains and spectra used for the database construction^48^. All *M. chelonae* isolates were correctly identified and well discriminated from the *M. abscessus* complex. As we only tested a limited number of *M. chelonae* isolates, more strains will be necessary for further confirmation. Other rarely isolated species, such as *M. mucogenicum*, *M. mageritense*, and *M. vaccae*, were well identified using VITEK MS V3.0. However, the limited number of isolates used in the present study was not enough to sufficiently evaluate the performance of these species using MALDI-TOF MS.

The present study was associated with a number of limitations. First, despite the large number of clinical *Mycobacterium* isolates included in the present study, almost all isolates were from respiratory tract samples, and a limited number of species were recovered. Second, only a few isolates were tested for some species, and a more thorough evaluation would seem worthwhile with more isolates collected globally. Third, we did not examine the identification performances of *Mycobacterium* species from other liquid media and thus cannot draw any conclusions regarding the performance of other isolation media. In follow-up studies, we plan to identify *Mycobacterium* directly from positive MGIT broth cultures to obtain a more comprehensive evaluation of MALDI-TOF MS used for *Mycobacterium* identification.

Nevertheless, the present study had several strengths. First, the present study evaluated the performance of the identification of a large number of clinical *Mycobacterium* isolates using the CE/IVD and FDA-approved VITEK MS Version 3.0 database. Second, the combined use of the VITEK MS *Mycobacterium/Nocardia* sample processing kit in *Mycobacterium* inactivation and protein extraction generated sample spectra compatible with the reference spectra provided by the manufacturer. With the process presented here, the identification performance was slightly higher than those reported by other researchers using the VITEK MS Version 3.0 database^28,29^.

In summary, we showed that *Mycobacterium* spp. can be adequately identified by the VITEK MS Version 3.0 database. The use of a commercial sample processing kit and standard protocols are likely to yield highly reliable results in the identification of *Mycobacterium* species. This study further confirmed that the implementation of MALDI-TOF MS in the clinical laboratory enables the highly accurate identification of *Mycobacterium* species.

## Materials and methods

### Clinical isolates

A total of 507 *Mycobacterium* isolates representing 28 species, were enrolled in the present study. Clinical samples were submitted to the Shanghai Pulmonary Hospital for mycobacterial culture, and these isolates were recovered between June 2015 and March 2017. For MALDI-TOF MS analysis, all isolates were subcultured on Löwenstein-Jensen (LJ) medium at 35 °C. Rapidly growing species were tested after 3 to 10 d of incubation, while slowly growing species were tested after 3 to 5 weeks of incubation at 35 °C.

### Viability assay

Inactivation was performed as previously described^17,21,31^. Briefly, colonies were selected from LJ medium with a 1-µL loopful of the organisms and then suspended in 1 mL of 75% ethanol and vortexed at maximum speed on a vortex adapter (Vortex-Genie 2, Scientific Industries, Inc. Bohemia, NY, USA) for 15 min followed by incubation for another 10 min at room temperature for inactivation. After centrifugation, the pellet was resuspended in 200 µL of sterile water, and the supernatant was discarded. Viability was measured by inoculating samples (100 µL) in BACTEC MGIT 960 bottles (BD Diagnostics, Shanghai, China) and subsequently assessing for growth on LJ medium. The LJ medium was examined daily for growth during the first week and then weekly thereafter, for a total of 6 weeks under incubation^28^. Viability was tested using the following strains: four isolates of *M. tuberculosis* and six NTM isolates (one each of *M. abscessus, M. fortuitum, M. kansasii, M. intracellulare, M. avium and M. lentiflavum*).

### VITEK^®^ MS *Mycobacterium/Nocardia* Kit

All procedures were performed with the VITEK® MS *Mycobacterium/Nocardia* Kit (bioMérieux SA, Marcy L’Étoile, France). In brief, a 1-µL loopful of organisms was picked and transferred into a tube with glass beads and 70% ethanol, followed by vortexing for 15 min and incubation at room temperature for another 10 min. After the subsequent transfer and centrifugation, the dried pellets were uniformly dispersed with 70% formic acid and acetonitrile in turn. After centrifugation, 1 µL of the supernatant was transferred to the target slide. Once the sample was dry, 1 µL of CHCA matrix (bioMérieux SA, Marcy L’Eoile, France) was applied and allowed to dry prior to analysis. All slides were run within 3 h after preparation, and the identifications were run in automatic mode. For each isolate, a single extraction was performed and spotted in duplicate. For any isolates where discrepancies were observed, the isolate was confirmed by gene sequencing (16 S *rRNA*, *hsp65* PRA or *erm*). For each run, *Escherichia coli* (ATCC 8739; American Type Culture Collection, Manassas, VA, USA) was used for calibration and quality control according to the manufacturer’s protocols. *Mycobacterium abscessus* ATCC 19977 was used as a positive control, while negative controls were simultaneously analyzed, which was performed with spotted matrix only^49^.

### VITEK MS knowledge base version 3.0 and MALDI-TOF MS analysis

Isolates were tested using the VITEK MS system (bioMérieux SA, Marcy L’Eoile, France), and the results were analyzed using knowledge base (KB) Version 3.0. Version 3.0 KB includes 49 *Mycobacterium* species grouped into 39 taxa, 14 *Nocardia* species, 48 mold species grouped into 47 taxa and 197 other new bacteria and yeasts, most of which are clinically relevant. Among the added 49 *Mycobacterium* species, there are 45 NTM (including 23 SGM and 22 RGM) and 4 MTBC species^29^. During the present analysis, if the identification score was lower than 99%, then reanalysis was performed, or the extraction was repeated. Repeat testing was performed with the original culture or a newly sub-cultured isolate, according to the procedures specified above. If an isolate still yielded no identification, then this sample was considered as an unidentified result^36^. For results in which the confidence score was lower than 50%, repeated extraction analysis and sequencing were performed.

### Sequencing

All analyzed strains were sequenced across the full 16 S *rRNA* gene using the universal primers 27-FOR (AGAGTTTGATCMTGGCTCAG) and 1492-REV (TACGGYTACCTTGTTACGACTT). For species such as *M. kansasii* and *M. gastri*, *M. fortuitum* group, *M. peregrinum* and *M. septicum*, *hsp65* PRA genes (FOR:ACCAACGATGGTGTGTCCAT and REV:CTTGTCGAACCGCATACCCT) were used to supplement the identification^50,51^. The *erm* gene was used for differentiating between *Mycobacterium abscessus* subspecies *abscessus*, *bolletii* and *massiliense*^45^. If the isolate identification obtained by VITEKVITEK MS Knowledge Base Version 3.0 was consistent with the gene sequence, then the result was considered as correct. For discrepancies between sequencing and MALDI-TOF MS analysis, the sequencing result was considered the gold standard.
